# Developmental profiling of gene expression in soybean trifoliate leaves and cotyledons

**DOI:** 10.1186/s12870-015-0553-y

**Published:** 2015-07-03

**Authors:** Anne V. Brown, Karen A. Hudson

**Affiliations:** Department of Agronomy, Purdue University, 915 West State Street, West Lafayette, IN 47907 USA; USDA-ARS Crop Production and Pest Control Research Unit, 915 West State Street, West Lafayette, IN 47907 USA

**Keywords:** Leaf senescence, Cotyledon senescence, *Glycine max*, Leaf development

## Abstract

**Background:**

Immediately following germination, the developing soybean seedling relies on the nutrient reserves stored in the cotyledons to sustain heterotrophic growth. During the seed filling period, developing seeds rely on the transport of nutrients from the trifoliate leaves. In soybean, both cotyledons and leaves develop the capacity for photosynthesis, and subsequently senesce and abscise once their function has ended. Before this occurs, the nutrients they contain are mobilized and transported to other parts of the plant. These processes are carefully orchestrated by genetic regulation throughout the development of the leaf or cotyledon.

**Results:**

To identify genes involved in the processes of leaf or cotyledon development and senescence in soybean, we used RNA-seq to profile multiple stages of cotyledon and leaf tissues. Differentially expressed genes between stages of leaf or cotyledon development were determined, major patterns of gene expression were defined, and shared genes were identified. Over 38,000 transcripts were expressed during the course of leaf and cotyledon development. Of those transcripts, 5,000 were expressed in a tissue specific pattern. Of the genes that were differentially expressed between both later stage tissues, 90 % had the same direction of change, suggesting that the mechanisms of senescence are conserved between tissues. Analysis of the enrichment of biological functions within genes sharing common expression profiles highlights the main processes occurring within these defined temporal windows of leaf and cotyledon development. Over 1,000 genes were identified with predicted regulatory functions that may have a role in control of leaf or cotyledon senescence.

**Conclusions:**

The process of leaf and cotyledon development can be divided into distinct stages characterized by the expression of specific gene sets. The importance of the WRKY, NAC, and GRAS family transcription factors as major regulators of plant senescence is confirmed for both soybean leaf and cotyledon tissues. These results help validate functional annotation for soybean genes and promoters.

**Electronic supplementary material:**

The online version of this article (doi:10.1186/s12870-015-0553-y) contains supplementary material, which is available to authorized users.

## Background

Soybean (*Glycine max*) is the world’s largest oilseed crop with many uses including oil, food, and biofuels. In 2013 over 75 million acres of soybean were grown in the US, for a total crop value of 42 billion dollars [1]. The completion of the soybean genome sequence in 2010 has accelerated progress in understanding the genetic control of many processes, as well as enabling functional genomics research towards crop improvement [2].

A leaf undergoes several important changes throughout its development. As the newly initiated leaf expands and gains photosynthetic competency, it undergoes a transition from nutrient sink to source, and later it undergoes senescence as nutrients are mobilized and exported prior to leaf abscission and a highly controlled process of cell death. During trifoliate leaf senescence in soybean, the nutrients that remain in the are transported to the developing seeds, therefore this process is crucial to yield and seed quality. If leaves senesce before seed development is complete (for example, as a result of a biotic stress in the form of foliar disease, or abiotic stress such as drought), the plant may suffer yield reductions up to 50 % [3], while senescence delay and extension of the seed filling period results in potentially increased yield [4]. Studies have also shown that a suppressed leaf senescence during water-deficient conditions can confer drought tolerance to tobacco plants [5]. Further understanding of the pattern of gene expression that is triggered during leaf senescence and how the initiation of leaf death is controlled presents an opportunity to maximize the photosynthetic period and potentially improve crop yields.

The cotyledon, a modified leaf that functions to sustain early seedling development follows a similar pattern, at least in soybean which undergoes epigeal phanerocotylar germination. Even among other legume species the cotyledon has a different fate – *Pisum* and *Phaseolus* cotyledons function exclusively as storage organs and do not attain photosynthetic competence, while in *Ricinius communis* the cotyledons lack the fleshy characteristics of soybean or common bean cotyledons and much more closely resemble leaves [6]. Establishment of the photosynthetic machinery in the soybean cotyledon is energetically costly for relatively little gain; it is thought that photosynthesis by soybean seedling cotyledons serves to balance respiratory losses soon after germination while the carbohydrate, lipid, and protein reserves are mobilized [7].

Previous expression profiling studies have identified genes associated with leaf development and senescence in the model plants *Arabidopsis* and maize and have further defined processes that occur during this period [8–10]. Genes that are up-regulated during senescence, termed senescence-associated genes (SAGs) encode proteins required for degradation, but which have roles in a variety of processes including defense responses, detoxification, and signaling. Genes that decrease in expression during senescence, referred to as senescence down-regulated genes (SDGs) primarily encode proteins involved with photosynthesis [11–13]. Along with SAGs and SDGs a number of transcription factors have been identified in various species that are implicated in the regulation of SAGs and SDGs [14–16]. The largest families of transcription factors associated with senescence include NAC, WRKY, MYB, C2H2 zinc finger, bZIP and AP2/EREBP [14, 15]. Some of these transcription factors have also been identified as highly expressed in the early seedling cotyledons [17]. Plant hormones play an important role in plant growth and development and also in the regulation of senescence. The salicylic acid, jasmonic acid and ethylene response pathways are known to be involved with plant defense responses and also positively regulate the senescence process, while cytokinins inhibit senescence [18, 19]. Studies in *Arabidopsis* have shown that mutants defective in salicylic acid signaling exhibit a delayed senescence and all three pathways are required for the expression of SAGs [20].

To characterize the temporal shifts in gene expression that underlie the biochemical and metabolic processes occurring in soybean leaves and cotyledons, and to identify the genes that govern these transitions, we performed RNAseq expression profiling on soybean leaves and cotyledons at multiple stages of the life cycle of each organ, including the stages before and during senescence. To more fully understand the mobilization of nutrient reserves in the cotyledon, we also examined the common and distinct processes between these two photosynthetic tissues.

## Results and discussion

### Stages and genes

Five stages were selected for profiling from the leaves from leaf opening to onset of senescence, which we refer to as L-I through L-V throughout this experiment. Three stages of development were analyzed from soybean cotyledons, which we refer to as C-I through C-III. The stages of leaf and cotyledon samples collected are shown in Additional files 1 and 2, respectively. Principal component analysis plots for leaf and cotyledon samples show consistency between biological replicates and significant differences between the stages selected for comparative analysis (Additional file 3). Stages L-II and L-III as well as stages L-IV and L-V are similar to one another, but very different from stage L-I. Principal component analysis for the cotyledon data suggests that gene expression in stages C-II and C-III is overall more similar than between stages C-I (immediately post-emergence) and C-II. A total of 38,079 (70.2 %) transcripts from the Gmax189 version of the soybean genome annotation were found to be expressed during at least one stage of leaf development, while 39,039 transcripts (72.1 %) were found to be expressed during at least one stage of cotyledon development (Additional files 4 and 5).

### Genes expressed differentially during development and senescence

**Fig. 1:**
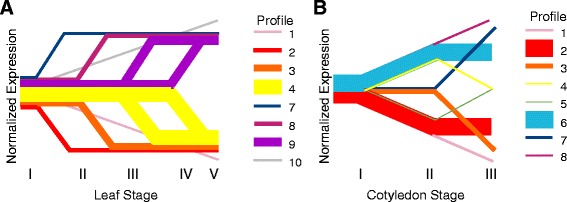
Differentially expressed gene profiles in leaf and cotyledon development. Differentially expressed genes from (**a**) leaves and (**b**) cotyledons were classified into expression profiles based on significant changes in gene expression between subsequent stages. Line thickness is proportional to the number of genes included in each profile. See Methods for more detail

Of the 54,175 genes in the soybean Gmax189 dataset, 12,497 genes (23.1 %) were found to be differentially expressed between at least two stages within the cotyledon timecourse while 9,500 genes (17.5 %) were differentially expressed between two stages of the leaf timecourse. Each differentially expressed gene was assigned to an expression profile based on significant fold change and expression across all of the stages in an organ order to identify major dynamic patterns of gene expression (Fig. 1). Overall, the set of differentially expressed genes through cotyledon and leaf development agree well with results of prior studies of leaf senescence in *Arabidopsis thaliana* and other plants. Close homologs for thirty-one percent (31 %) of genes identified as SAGs in *Arabidopsis* [13] were found to be differentially expressed in the leaf dataset, while 44 % were differentially expressed in the cotyledon dataset (Additional file 6). For example, *Arabidopsis* gene AtNAP (At1g69490) is a NAC transcription factor that has an important role in leaf senescence [21]. The top soybean orthologs of this gene are Glyma13g35550, Glyma1g06150, Glyma20g04400, Glyma02g12220, and Glyma07g35630. All of these were found to be significantly upregulated in either cotyledon or leaf in this experiment suggesting conservation of function of this transcription factor in soybean leaf senescence.

### Temporal pattern of leaf gene expression

**Fig. 2:**
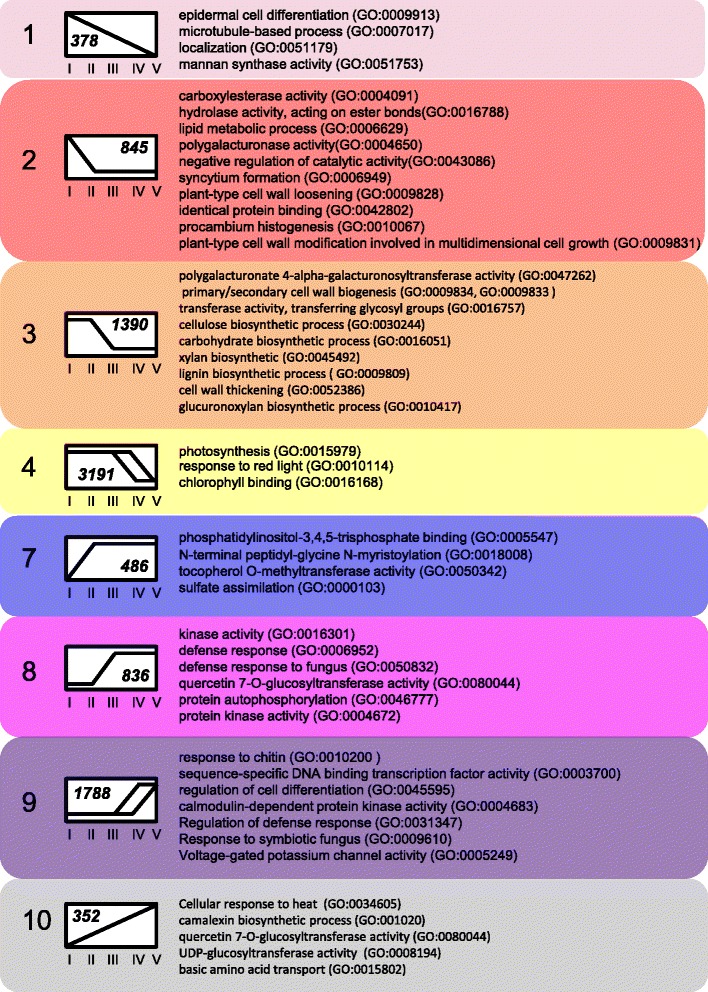
Significant GO term associations in leaf developmental patterns. Gene Ontology (GO) terms that were significantly enriched in the identified gene expression profiles from developing leaves are shown. The number of genes included in each profile is shown in the box that represents the expression pattern of the included genes. A full listing of significant GO-terms can be found in Additional file 7

**Fig. 3:**
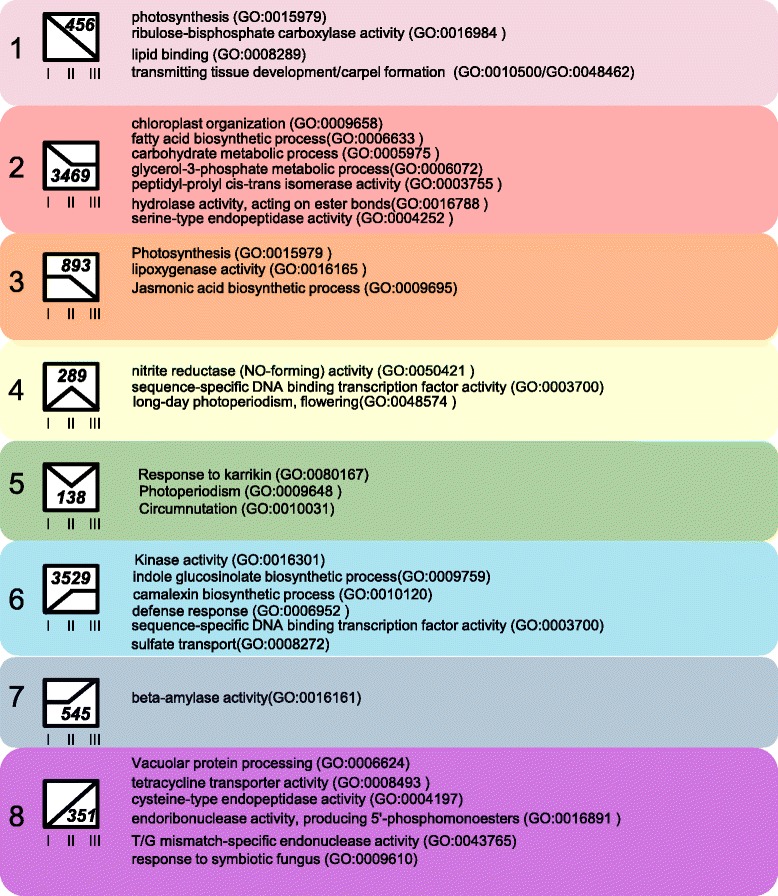
Significant GO associations in cotyledon development. Gene Ontology (GO) terms that were significantly enriched in the identified gene expression profiles from the cotyledon gene expression timecourse are shown. The number of genes included in each profile is shown in the box that represents the expression of the included genes. Full listing of significant GO-terms can be found in Additional file 7

To characterize the biochemical and physiological processes occurring during different stages of leaf development, we used Gene Ontology Term (GO-term) enrichment analysis [22] to identify groups of genes involved in similar functions that were significantly enriched within the co-regulated gene sets that were identified. A table listing all enriched GO-terms and associated genes identified in each leaf or cotyledon expression profile is given in Additional file 7, and summarized in Figs. 2 & 3. Genes belonging to profile 2 or 3 that decreased significantly between stage L-I and L-II or L-III fell into several classes, predominantly associated with cell growth and expansion, cell wall biosynthesis, cell wall thickening and loosening, as well as the development of the epidermal cell layer and cuticular wax biosynthesis (Fig. 2). This finding is consistent with the L-I stage being critical for leaf expansion and maturation (Leaves are fully expanded by stage L-II). Genes associated with photosynthetic processes and light response decreased in expression during the later stages of L-IV and L-V (profile 4) consistent with the decline of photosynthesis as the tissues began to senesce. Genes expressed at higher levels in the later stages (profile 10) function in arginine and glutamate transport, consistent with remobilization of amino acids (nitrogen) from the senescing tissues. Seven genes with predicted roles in sulfate assimilation are upregulated early (profile 7) which is consistent with the previously observed developmental regulation of the ATP sulfurylase genes [23]. A significant number of genes potentially involved in disease resistance and defense responses were also upregulated after stage L-III, which is consistent with the overlapping functions of many senescence-associated genes in responses to biotic stress (reviewed in [24]).

### Temporal pattern of cotyledon gene expression

Genes that increased in expression between stages C-I and C-III (profile 8) or C-II and C-III (profile 7) were implicated in protein and starch breakdown (Fig. 3). Genes in profiles 4 and 6 that increase in expression between stage C-I and C-II have roles in defense response, nitrate assimilation, and sulfate transport. A large number (3469, or approximately 10 % of the total number of genes observed to be expressed in cotyledons) decreased in expression between stages C-I and C-II (profile 2) and these genes appear to function in lipid and carbohydrate metabolism and proteolysis, consistent with the role of cotyledons in mobilizing reserves to the developing seedling shortly after germination [17]. Some of the changes in gene expression observed between C-I and C-II may be due to the differences in the growth conditions for these tissues, however we can infer that the genes that respond in a similar pattern in L-I and L-III may be developmentally rather than environmentally induced. Genes that decrease in stage C-III (profiles 1 and 3) are primarily involved in the biosynthesis and maintenance of the photosynthetic complex, and jasmonic acid biosynthesis.

**Fig. 4:**
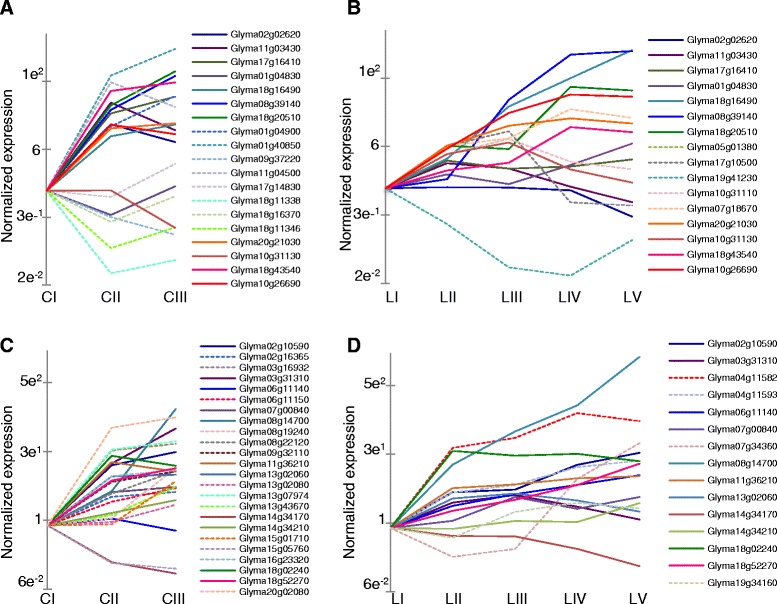
Regulation of N and S transporter genes. Differentially expressed genes annotated as N transporters in (**a**) cotyledons and (**b**) leaves. Blue and green lines represent nitrate transporters, pink and red lines represent ammonium transporters. Solid lines represent genes that were differentially expressed in both tissues, while dashed lines show genes that were differentially expressed in either cotyledons or leaves. Genes annotated as sulfate transporters from the cotyledons are shown in (**c**) and from the leaves in (**d**) Y-axis is log_2_ scale

A significant number of genes included in cotyledon profile 6 (upregulated early) are annotated as sulfate transporters. Nitrogen (N), Potassium (K), Phosphorus (P), and Sulfur (S) are major nutrients mobilized from yellowing leaves to new growth or developing seeds. It was shown that N, P, K, and S levels drop dramatically during *Arabidopsis* leaf senescence, with a loss of over 60 % of the initial nutrient content [25]. To understand the role of transcriptional regulation in the process of nutrient mobilization, we examined the response of the all of genes annotated as transporters of nitrogen, sulfur, potassium, or phosphorus. Approximately half of the annotated or BLASTP-identified N transport genes were expressed at levels below the threshold of detection of this experiment, but many of the transporter genes expressed at high levels were identified as differentially regulated. These genes are listed in Additional file 8. Fig. 4 shows the differentially expressed N (ammonium and nitrate) and S transporters in the cotyledon and leaves. The upregulated nitrate transporters predominantly belong to the NPF2, NPF4, and NPF7 families, with an affinity for nitrate or dipeptide transport, and related to the Arabidopsis NRT1.7 with a role in source-sink remobilization of N [26, 27]. Consistent with results from *Arabidopsis*, nitrogen and sulfate transporter genes are expressed at higher levels in the later stages of both the leaves and cotyledon. Of 39 transcript models annotated as sulfate transporters, 22 were upregulated between stages C-I and C-II in cotyledons, and 14 were significantly upregulated in leaf tissues, and eleven of these were common to both sets. N transporters were also upregulated in both leaves and cotyledons. Potassium and phosphate transporters were differentially expressed during leaf and cotyledon development and senescence, but demonstrated less coordinated regulation (Additional file 8).

### Modifications of the leaf expression blueprint to program a nutrient reserve

**Fig. 5:**
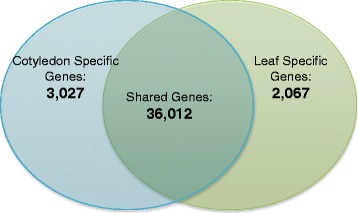
Leaf and cotyledon specific genes. Of the 41,106 genes identified as expressed in the experiment, 7.4 % of genes were expressed exclusively in the cotyledons, 5 % of the genes were expressed only in the leaf, and more than 87 % were shared between the two tissues. The list complete list of genes that demonstrated tissue specific expression is included in Additional file 9

We determined that 36,012 transcripts (87.6 %) were expressed in both the cotyledon and the leaf, while the remainder of the genes showed evidence for specificity to either leaves or cotyledons in this experiment (Additional file 9). A total of 3,027 (7.4 %) genes were only present in cotyledons and 2,067 genes (5.0 %) were only expressed in the leaf (Fig. 5). Over-represented GO-terms associated with the cotyledon-specific genes suggest the mobilization of cotyledon-specific nutrients, including the response to trehalose stimulus and alpha-glucan/water dikinase activity. The chloroplast protein alpha-glucan water dikinase 1 mediates the incorporation of phosphate into starch-like alpha-glucan, mostly at the C-6 position of glucose units in *Arabidopsis.* This acts as an overall regulator of starch mobilization, and is required for starch degradation [28]. The β**-**amylase enzymes are involved in the hydrolysis of starch and an important step in germinating seedlings, and post germination growth [29, 30]. Over-represented functions for the leaf-specific genes include categories associated with photoperiodism and flower development, consistent with these processes occurring long after the cotyledons have senesced.

Genes expressed in both leaf and cotyledon tissues, and which demonstrated significant differential expression between both C-II and C-III and L-III and L-IV (genes that change in expression in anticipation of tissue senescence in both organs) were shown to have a significant overlap, and 99.0 % of these genes shared the same direction of change (up- or down-regulation) in both tissues. This may indicate that the control of the later phases of senescence uses the same molecular machinery in both tissues (Additional file 10). Genes expressed differentially between early stages, as between both C-I and C-II and L-I and L-II also overlapped significantly and over 95 % of these genes shared the same direction of change, which likewise implies a shared mechanism for early growth and developmental patterning common to both tissue types.

### The control network of temporal gene expression

One of the largest classes of genes found to be differentially expressed between multiple stages were the transcription factors. To investigate regulatory networks involved with developmental processes we identified transcription factors that were differentially expressed throughout the time course. We assembled a list of 7,251 transcription factors from the soybean genome based on Mapman classification terms [31]. In leaves, a total of 990 genes predicted to encode transcription factors were differentially expressed between two stages. A total of 1415 transcription factors were differentially expressed between at least two stages of cotyledon development.

**Fig. 6:**
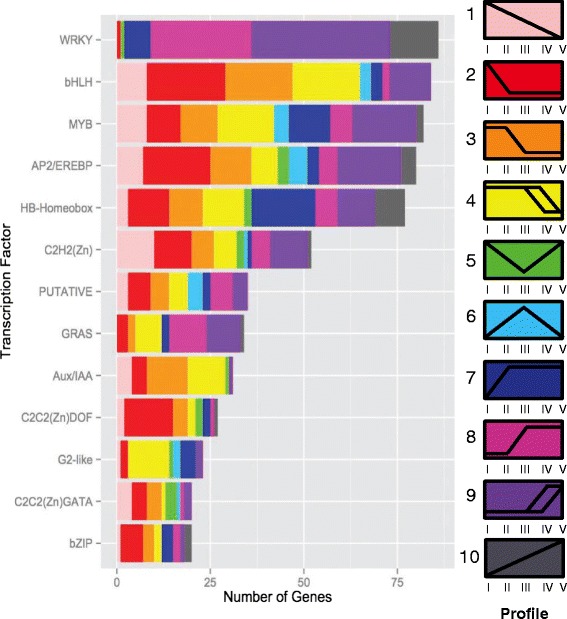
Specific families of leaf transcription factors are enriched in early leaf development and senescence phases. Differentially expressed transcription factors categorized by expression profile and classified by family. Transcription factor families represented by more than twenty genes are shown [31]

**Fig. 7:**
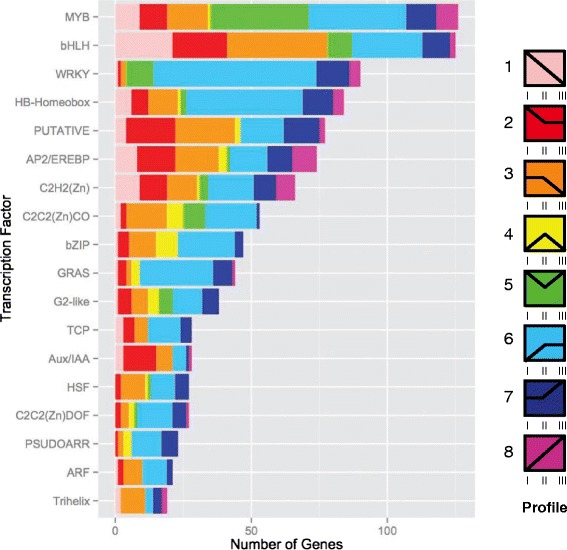
Specific families of transcription factors are enriched in different phases of cotyledon post-germination development. Differentially expressed transcription factors categorized by expression profile and classified by family. Transcription factor families represented by more than twenty genes are shown [31]

The transcription factors from both the cotyledon and leaves were placed into bins based on annotated family [31]. We found representatives of 60 different families of transcription factors in the cotyledon and 57 families in the leaf. The most abundant transcription factor families (containing 20 or more genes) are shown in Fig. 6 for leaves and Fig. 7 for cotyledons. In both tissues, we observe that the transcription factors most commonly found to increase in expression with tissue age are from the WRKY family and these comprise the largest family of transcriptional regulators expressed differentially in our dataset. This is consistent with previous studies that find these transcription factors to function as major regulators of senescence [29, 32–34]. Additionally, members of the GRAS family are also expressed predominantly during leaf senescence and early cotyledon senescence, consistent with studies in other species [35–37]. Twenty-one of the GRAS transcription factors were upregulated during senescence in both cotyledons and leaves. In leaves, the bHLH, C2C2(DOF) zinc finger transcription factors, and G2-like transcription factors share the expression profiles (profiles 2–4) that span the photosynthetic period, consistent with a likely role in chloroplast development and regulation of photosynthetic genes and carbohydrate metabolism [38–41]. Auxin plays an important role in plant growth and development by regulating gene expression [42]. In both the leaf and cotyledon, the Aux/IAA transcription factors decrease in expression following the initial stages of development (leaf profile 3 and cotyledon profile 2). This result is consistent with studies of senescence in cotton, rice, and wheat suggesting these transcription factors may act as negative regulators of senescence [43–45]. Of 7,251 genes identified by MapMan as transcription factors, only six were annotated as part of the NAC family. As these transcription factors are known to be involved in senescence and defense [14, 21, 46], we examined the Gmax_189 genome annotations to identify 236 NAC or NAC-related transcription factors (Additional file 11), 38 of these were found to differentially expressed in the leaves and 54 in the cotyledon. The majority of the differentially expressed NAC transcription factors (23 in leaf and 40 in cotyledon) were upregulated during tissue senescence, with 8 genes common to both organs.

**Fig. 8:**
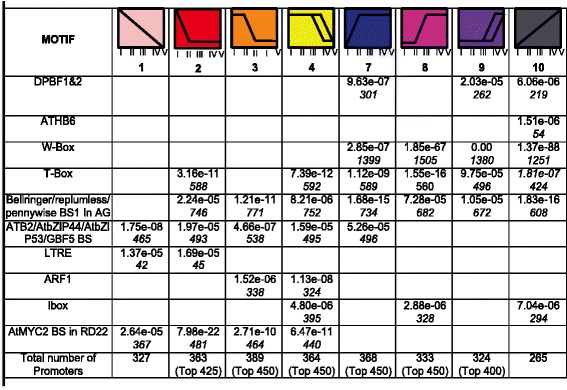
Significantly over-represented motifs in promoters of genes that change in expression during leaf development and senescence. The table shows the significant promoter motifs (*e*-value cutoff less than *e*-05) and the number of elements (in italics) found in the set of promoters of genes classified in one of the six expression profiles [47]. For leaf analysis, multiple profiles were combined to identify significant motifs. Leaf profiles 2 and 3 were combined, while profile 4 was pooled with genes decreasing in stage L-V. Profiles 5 and 6 did not include a sufficient number of genes to identify significant motifs. Profiles 7 and 8 were pooled into one profile. Profile 9 included additional genes that increased in expression between stages L-IV and L-V. Where gene sets were too large for analysis, the top 425 or 450 genes (based on magnitude of fold change) were analyzed

**Fig. 9:**
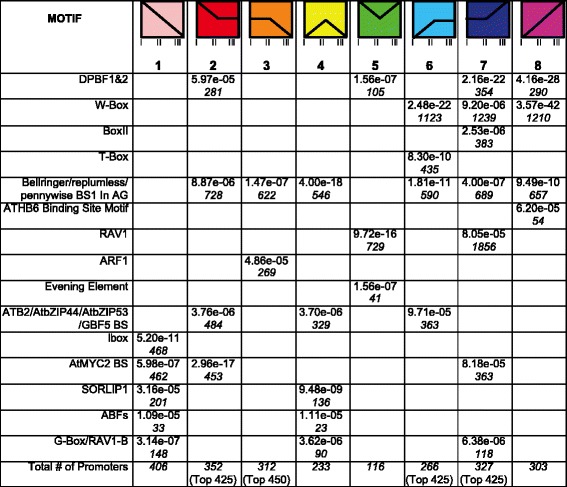
Significantly over-represented motifs in promoters of genes that change in expression during cotyledon senescence. The table shows the most significant promoter motifs (*e*-value cutoff less than *e*-05) and the number of occurrences (in italics) for each significant element found in promoters of genes classified in one of the eight expression profiles identified for differentially expressed cotyledon genes [47]. Where gene sets were too large for analysis, the top 425 or 450 genes (based on magnitude of fold change) were analyzed

To identify potential *cis*-acting elements that govern the temporal patterns of expression, over-represented promoter motifs were identified from genes in each profile using Elefinder [47, 48]. The Elefinder method identifies statistically over-represented, characterized binding sites for known transcription factors from a variety of plant species within a specified promoter set. This can suggest a likely mechanism for the control of the specific expression pattern of a gene although the incidence of a given binding site only suggests which class of transcription factor could recognize and regulate the promoter. Where gene sets were too large to scan for over-represented elements in their entirety, the top 400,425 or 450 genes (based on largest fold change) were analyzed. Fig. 8 lists all of the significant motifs associated with each expression pattern in leaves, and the associated *e*-value, and the number of occurrences of the element in the data set. In the leaf, elements present in the promoters of genes that decline from stage L-I throughout the timecourse (leaf profiles 1–4) are associated with photosynthetic genes, including the MYC2 binding site, the Ibox, and ATB2/AtbZIP44/AtbZIP53/GBF5 binding site. Similarly, genes included in cotyledon profiles 1 and 4 that decreased in expression between stages C-II and C-III contained the G-box and SORLIP1 element in their promoters, consistent with the known abundance of these motifs upstream of light regulated genes associated with photosynthesis. Photosynthesis genes were observed to be downregulated between these stages (Fig. 8), thus activation via these elements may be reduced during the studied period [47, 49, 50]. The ABFs element is enriched in promoters of genes declining in expression throughout the cotyledon timecourse, and is a G-box variant implicated in ABA response and targeted by bZIP transcription factors [51] (Fig. 9). The ARF1 binding site, targeted by the Aux/IAA transcription factors is enriched in genes downregulated in later stages of development in both the leaf (profiles 3 and 4) and cotyledon (profile 3) [52, 53]. In both leaves and cotyledons, the DPBF1&2 element is enriched in genes upregulated in later stages (for example, profiles 9 and 10 in the leaf and 5, 7, and 8 in the cotyledon) [54–56]. In both leaves and cotyledons, the AtHB6 motif is over-represented in genes that had a constant increase in expression (cotyledon profile 8 and leaf profile 10). The ATHB6 transcription factor is a member of the homeodomain-leucine zipper family of transcription factors thought to regulate active cell growth and differentiation (reviewed in [57]). The Bellringer/replumless/pennywise binding site is enriched in promoters of genes from multiple distinct expression profiles in cotyledons and leaves. This AT-rich *cis*-regulatory element is a target for homeodomain transcription factors, however in the context of regulation of the AGAMOUS gene in *Arabidopsis* the element is located within an intron [58]. In both tissues the WRKY-targeted W-box [14, 59] is the most significantly enriched promoter element, and occurs in genes that increase in expression leading up to leaf senescence.

Transcriptional control mechanisms leading to differential gene expression are believed to play important roles in coordination of the senescence process [14]. Our results support previous findings that major transcription factor families (eg, WRKY) are associated with senescence. The WRKY family is one of the largest transcription factor families in higher plants and 133 WRKY members have been identified in the soybean genome [60]. Eighty-six WRKY transcription factors were differentially expressed in the leaf, and 91 in the cotyledon. Of these WRKY genes, 44 were differentially expressed in both leaves and cotyledons, indicating that the WRKY transcription factors play a major role in regulation in both tissues. Supporting the hypothesis that the co-regulated sets of genes expressed in distinct stages of cotyledon development share a mechanism of regulation, it was found that the W-box (the binding site for WRKY transcription factors) has by far the most significant over-representation in the promoters of genes that increase in expression during senescence in both cotyledon and leaf tissues. These results are consistent with the hypothesis that these master regulators lie at the top of a network driving the expression of genes that drive the physiological changes observable during senescence.

## Conclusions

We have profiled the temporal and developmental regulation of approximately 41,000 genes in trifoliate leaves and cotyledons of soybean. We found several commonalities between these two tissues for the establishment of photosynthetic machinery, and in the final processes of mobilization of nutrients, but have also identified specific transcripts that appear to be involved in mobilization of stored nutrients in the cotyledons and are expressed earlier in cotyledon development. Significantly, we have identified families of transcriptional regulators and corresponding *cis*-regulatory motifs that appear to co-ordinately regulate the changes in expression observed in several groups of genes during the programmed transition to senescence. Our data help to identify the specific machinery by which the genome is regulated during the later stages of leaf development, and may allow more specific biotechnology approaches targeting developmental stages and tissues with transgene expression.

## Methods

### Plant growth and tissue collection

Williams-82 soybean seeds were planted in one part Promix (BRK): two parts sand/soil min in 3-gal pots [61]. Plants were grown in Conviron PGR14 chambers in 12 hr light/12 hr dark photoperiod conditions in light provided by a combination of fluorescent and incandescent lighting at an intensity of 350 μmol m^−2^ s^−1^, constant temperature of 25 C and 75 % relative humidity. Williams-82 plants grown in these conditions flower after 6 weeks and produce seed. Samples were collected at 2:00 pm (6 hours after lights were turned on) to minimize diurnal effects. Cotyledon samples grown for stage C-I were grown in magenta boxes on MS media (without sucrose, MP Biomedicals, LLC, Solon, Ohio) to reduce the risk of soil contamination. Three biological replicate sets of cotyledons (one pair of cotyledons) were collected for each sample. Five distinct stages determined by physical appearance and plant developmental stage were collected from the time of soil emergence to the onset of senescence (see Additional file 2). Stage C-I cotyledons were collected immediately after emergence (4 days after planting) and were green but not yet open. Stage II cotyledons were open, green, and collected after the plant produced the first set of unifoliate leaves. Stage III cotyledons were collected at the first sign of yellowing and shrinking. Three biological replicate sets of leaves (one trifoliate) were collected from different individuals, at the 4th node (3rd set of trifoliate leaves) of each plant. Initial tissue collection of the sets of trifoliate leaves began at the V3 [62] stage and subsequently occurred every seven days. Eight stages were collected Stage L-I was collected after the trifoliates opened but before the leaves were fully expanded. Stages L-II and L-III were collected 21 and 40 days after stage L-I, respectively. Stages L-IV and L-V were collected after 49 and 56 days, and these two samples were marked by the appearance of signs of leaf senescence (Additional file 1). RNA was extracted using procedures described previously [61].

### Library preparation and sequencing

5 μg of RNA from each sample was used for library construction using standard protocols. Paired-end libraries were constructed for both leaf and cotyledon samples. Median insert size was 200 bp. Three biological replicate RNA samples from each stage of the cotyledons were sequenced using the Illumina Hi-Scan with three libraries per lane. The total number of mate-paired reads for each sample ranged from 5,000,000 to 54,000,000. Three biological replicate RNA samples from each stage of the leaves were sequenced in one lane on the Illumina Hi-Seq 2500, and the number of reads ranged from 5,000,000 to 18,000,000. For both leaves and cotyledons, read lengths of 100 bp were collected.

### Read alignment and normalization

Reads were aligned to the Glycine max transcriptome (Gmax_189) [2] using the ultrafast Bowtie aligner [63]. The alignment allowed only one mismatch per read. Approximately 87 % of reads matched known soybean transcript models. Of those reads 23 % matched only one transcript and 77 % matched multiple transcripts. (These figures were approximately the same for both leaf and cotyledon tissues.) Reads that hit more than one transcript were filtered from the bowtie output and only the reads that matched only one transcript were used for further analysis [64]. Read counts were obtained from the Bowtie output using a set of Perl scripts. Transcripts with less than 10 total read counts per gene in at least one stage were removed. The remaining transcripts were normalized for each sample by dividing the effective library size for each sample from the raw count of that sample (using the DESeq software package). These normalized counts were used for further analysis. Principal component analysis (PCA) was performed using the DESeq package, on the whole transcriptome set with low-expression genes removed. This method used variance-stabilized data to obtain sample-to-sample distance. To identify differentially expressed genes both DESeq and EdgeR software packages were used to perform pairwise comparisons of stages and for both packages with a corrected p-value of 0.05, and a 1.5 log_2_ fold (2.8 fold) change [65, 66]. The set of differentially expressed genes common to both of these two methods make up the set of genes used for further analysis. (Additional file 4). Differentially expressed genes from cotyledons were assigned to a profile using a script in R based on whether expression significantly increased or decreased between two stages, leaf genes were manually evaluated for inclusion into each profile. The majority of genes had either a downward or upward trend. Relatively few genes fell into profiles that initially decreased and then increased, or vice versa, the genes that exhibited the up-then-down pattern (profile 6, 70 genes) or down-then-up pattern (profile 5, 49 genes) were combined for promoter and GO-enrichment analysis. Genes that showed increased expression between more than two stages in leaves were also combined into the “overall up” (profile 10) and genes that decreased in expression between more than two stages were placed in the “overall down” profile (profile 1). Leaf profiles 4 and 9 also included distinct patterns, as stages L-IV and L-V were similar (see Additional file 3), genes that increased in expression between L-III and L-IV or between L-IV and L-V were pooled into profile 9, and genes whose expression decreased between L-III and L-IV or L-IV and L-V were pooled into profile 4.

A larger number of differentially expressed genes were found in the cotyledons relative to leaves, potentially due to higher sequence depth for the cotyledon samples.

### Data analysis

GO-term analysis was performed using the Soybase GO-enrichment tool (www.soybase.org) using GO annotation terms from genome version Gmax1.89. Promoter motif analysis was performed using the Elefinder tool at http://stan.cropsci.uiuc.edu/tools.php [48]. BLASTP was used to identify soybean orthologs of Arabidopsis SAGs from the TAIR10 version of the *Arabidopsis thaliana* genome annotation.

### Availability of supporting data

The raw and processed data sets supporting the results of this article are available in the NCBI Gene Expression Omnibus, as set GSE61857 at http://www.ncbi.nlm.nih.gov/geo/.

## Additional files

Additional file 1:
**Stages of leaf development.**
Additional file 2:
**Stages of cotyledon development.**
Additional file 3
**Principal component analysis of leaf and cotyledon gene expression.**
Additional file 4:
**Normalized count data for all genes in the leaf and cotyledon tissues.**
Additional file 5:
**Differentially expressed genes and normalized expression values for leaf and cotyledon developmental timecourses with annotations.**
Additional file 6:
**Normalized expression values of Homologs for Arabidopsis Senescence Associated Genes in leaf and cotyledon.**
Additional file 7:
**Enriched GO terms for co-expressed genes.**
Additional file 8:
**Normalized expression values of genes involved in nitrogen, sulfur, potassium, and phosphorus transport.**
Additional file 9:
**Tissue specific genes.**
Additional file 10:
**Differentially expressed genes common between leaf and cotyledon.**
Additional file 11:
**Normalized expression values of NAC annotated genes in leaf and cotlyedon.**

## Abbreviations

NAC: NAM, ATAF, CUC transcription factor
WRKY: WRKY motif transcription factor family
MYB: Myeloblastosis transcription factor family
ATHB6: *Arabidopsis thaliana* homeobox 6 transcription factor
C2H2 zinc finger: Cys(2)His(2) zinc finger motif transcription factor family
bZIP: Basic leucine zipper transcription factor
AP2/EREBP: APETALA2/ETHYLENE RESPONSE ELEMENT2 transcription factor
ABA: Abscisic acid
GRAS: GAI, RGA, SCR transcription factor family
bHLH: Basic helix-loop-helix transcription factor
C2C2(DOF): Cys(2)Cys(2) DNA binding with one finger zinc finger transcription factor
SORLIP: Sequence over-represented in light-induced promoters
DPBF: *Dc3* promoter binding factor recognition site
ARF: Auxin response factor
