# Supplementary material for: Developmental profiling of gene expression in soybean trifoliate leaves and cotyledons
Source: BMC Plant Biol. 2015 Jul 3;15:169. doi: 10.1186/s12870-015-0553-y (PMC4492100; doi:10.1186/s12870-015-0553-y)
Supplement: Additional file 1: — Stages of leaf development. [file 12870_2015_553_MOESM1_ESM.pdf]

Additional File 1. Stages of leaf development

L-I L-II

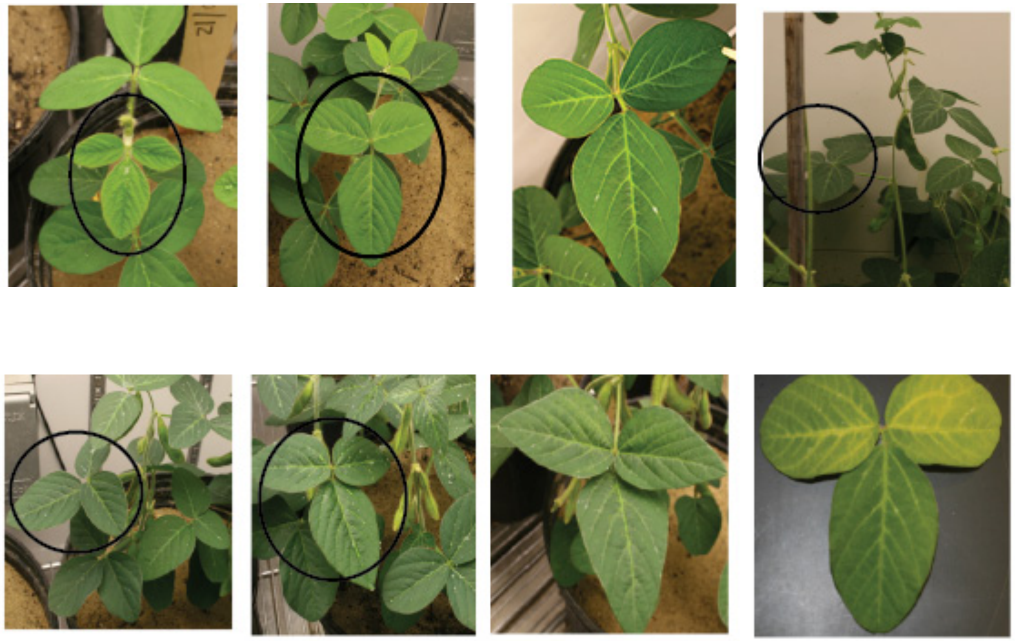

L-III L-IV L-V

Stages L-I, L-II, L-III, L-IV, and L-V were sent for sequencing. Intermediate stages are shown but were not used for RNAseq analysis.
