# Supplementary material for: Developmental profiling of gene expression in soybean trifoliate leaves and cotyledons
Source: BMC Plant Biol. 2015 Jul 3;15:169. doi: 10.1186/s12870-015-0553-y (PMC4492100; doi:10.1186/s12870-015-0553-y)
Supplement: Additional file 2: — Stages of cotyledon development. [file 12870_2015_553_MOESM2_ESM.pdf]

Additional File 2. Stages of cotyledon development

C-I

C-II

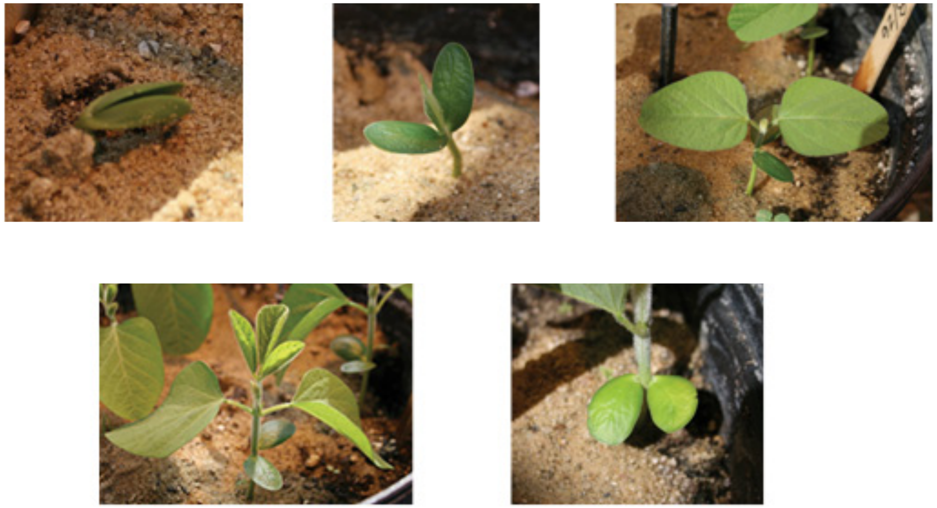

C-III

Stages C-I, C-II, and C-III were sent for sequencing. Intermediate stages are shown but were not used for RNAseq analysis.
