# Supplementary material for: Developmental profiling of gene expression in soybean trifoliate leaves and cotyledons
Source: BMC Plant Biol. 2015 Jul 3;15:169. doi: 10.1186/s12870-015-0553-y (PMC4492100; doi:10.1186/s12870-015-0553-y)
Supplement: Additional file 3 — Principal component analysis of leaf and cotyledon gene expression. [file 12870_2015_553_MOESM3_ESM.pdf]

Additional File 3.

A. Principal Component Plot – Cotyledon samples

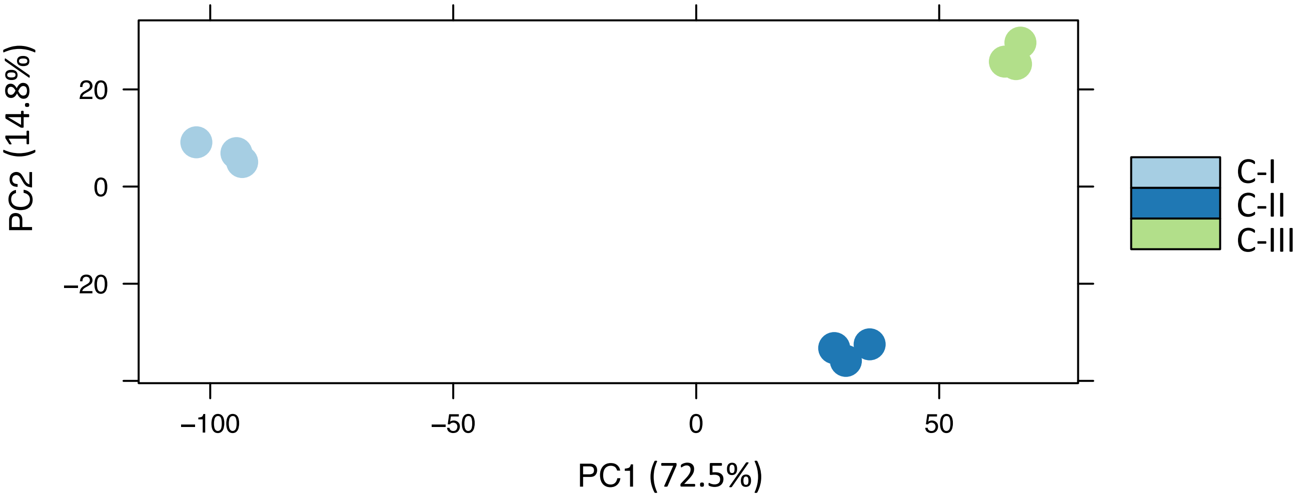

B. Principal Component Plot – Leaf samples

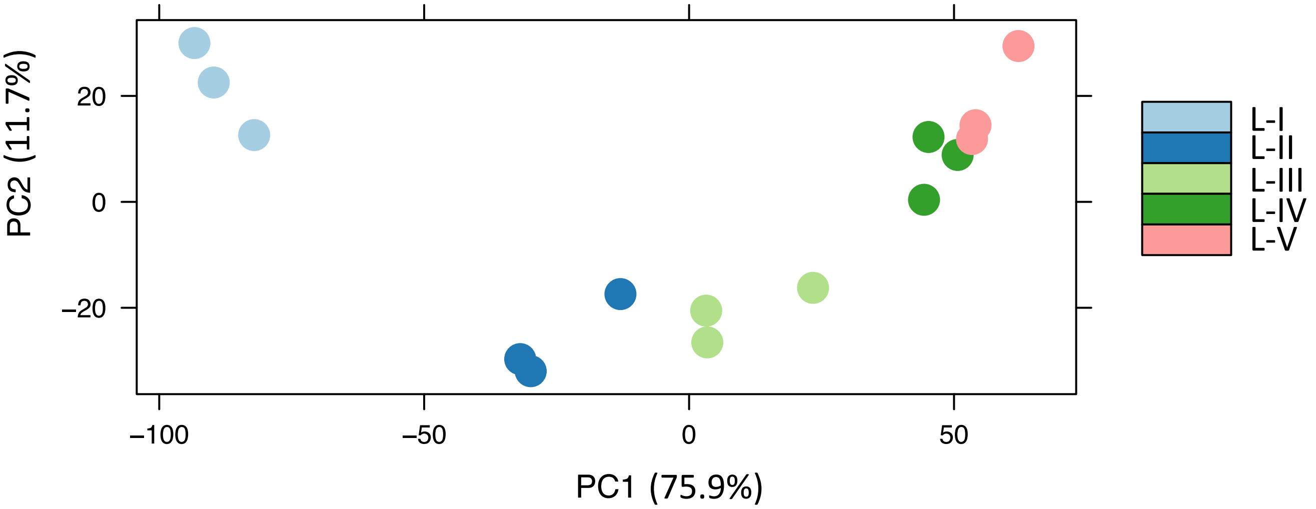

Principle Component Analysis was done using the DESeq package by calculating a variance stabilizing transformation from the fitted dispersion-mean relation and then transforming the normalized count data (Anders et al. 2010). The percent shown on the axes indicate the proportion of variance for that principal component.
