# Supplementary material for: Developmental profiling of gene expression in soybean trifoliate leaves and cotyledons
Source: BMC Plant Biol. 2015 Jul 3;15:169. doi: 10.1186/s12870-015-0553-y (PMC4492100; doi:10.1186/s12870-015-0553-y)
Supplement: Additional file 10: — Differentially expressed genes common between leaf and cotyledon. [file 12870_2015_553_MOESM10_ESM.pdf]

Additional File 10. Differentially expressed genes common between leaf and cotyledon

	CI-II	CI-III	CII-III	LI-II	LI-III	LI-IV	LI-V	LII-III	LII-IV	LII-V	LIII-IV	LIII-V	LIV-V
CI-II	9,484	7,640	1,365	1,030	2,159	3,282	3,499	164	1,097	1,375	133	404	10
CI-III		12,561	2,306	1,182	2,512	4,094	4,699	204	1,404	1,995	165	561	9
CII-III			2,983	278	620	1,103	1,372	63	470	864	80	332	4
LI-II				2,003	1,716	1,775	1,745	84	417	479	59	137	3
LI-III					4,306	3,896	3,789	283	1,178	1,318	119	303	6
LI-IV						7,479	6,240	292	2,098	2,330	244	622	12
LI-V							8,344	281	1,947	2,736	234	732	18
LII-III								326	303	284	38	60	1
LII-IV									2,463	1,884	248	500	6
LII-IV										3,306	237	752	20
LIII-IV											278	200	1
LIII-V												869	17
LIV-V													26

Numbers of genes differentially expressed between paired samples across the experiment. CI-II genes are differentially expressed between stages C-I and C-II, LI-LII genes are differentially expressed between stages L-I and L-II. In this case there are 1030 genes that belong to both sets.
